# Morphology and Hydraulic Architecture of *Vitis vinifera* L. cv. Syrah and Torrontés Riojano Plants Are Unaffected by Variations in Red to Far-Red Ratio

**DOI:** 10.1371/journal.pone.0167767

**Published:** 2016-12-02

**Authors:** Carina Verónica González, María Florencia Jofré, Hernán F. Vila, Markus Stoffel, Rubén Bottini, Carla Valeria Giordano

**Affiliations:** 1 IBAM (Instituto de Biología Agrícola de Mendoza), UNCuyo, CONICET. Facultad de Ciencias Agrarias. Almirante Brown 500 Chacras de Coria, Luján de Cuyo, Mendoza, Argentina; 2 Facultad de Ciencias Exactas y Naturales, Universidad Nacional de Cuyo. Padre Contreras, Mendoza, Argentina; 3 Laboratorio de Viticultura, Estación Experimental Agropecuaria Mendoza, Instituto Nacional de Tecnología Agropecuaria. San Martin 3853, Mayor Drummond, Luján de Cuyo, Mendoza, Argentina; 4 Dendrolab.ch, University of Berne, Baltzerstrasse 1+3, Berne, Switzerland; 5 Climatic Change and Climate Impacts, Institute for Environmental Sciences, University of Geneva, Boulevard Carl-Vogt 66, Geneva, Switzerland; 6 IADIZA (Instituto Argentino de Investigaciones en Zonas Áridas), CONICET. Av. Ruiz Leal s/n, Parque General San Martín, Mendoza, Argentina; UC Davis MIND Institute, UNITED STATES

## Abstract

Plants have evolved an array of specific photoreceptors to acclimate to the light environment. By sensing light signals, photoreceptors modulate plant morphology, carbon- and water-physiology, crop yield and quality of harvestable organs, among other responses. Many cultural practices and crop management decisions alter light quantity and quality perceived by plants cultivated in the field. Under full sunlight, phytochromes perceive high red to far red ratios (R:FR; 1.1), whereas overhead or lateral low R:FR (below 1.1) are sensed in the presence of plant shade or neighboring plants, respectively. Grapevine is one of the most important fruit crops in the world. To date, studies on grapevine response to light focused on different Photosynthetic Active Radiation (PAR) levels; however, limited data exist about its response to light quality. In this study we aimed to investigate morphological, biochemical, and hydraulic responses of *Vitis vinifera* to variations in R:FR. Therefore, we irradiated Syrah and Torrontés Riojano plants, grown in a glasshouse, with lateral FR light (low lateral R:FR treatment), while others, that were kept as controls, were not irradiated (ambient lateral R:FR treatment). In response to the low lateral R:FR treatment, grapevine plants did not display any of the SAS morphological markers (i.e. stem length, petiole length and angle, number of lateral shoots) in any of the cultivars assessed, despite an increase in gibberelins and auxin concentrations in leaf tissues. Low lateral R:FR did not affect dry matter partitioning, water-related traits (stomata density and index, wood anatomy), or water-related physiology (plant conductance, transpiration rate, stem hydraulic conductivity, stomatal conductance). None of the *Vitis vinifera* varieties assessed displayed the classical morphological and hydraulic responses associated to SAS induced by phytochromes. We discuss these results in the context of natural grapevine environment and agronomical relevance.

## Introduction

Solar radiation is a key source of energy for plant growth, as well as a source of signals sensed by plants that trigger growth, developmental and phenological responses for acclimation to the prevailing environment [1]. Plants respond to reductions in light availability mainly through two syndromes, shade tolerance or shade avoidance. Shade-tolerant plants display morphological and physiological traits that increase light interception and utilization, these include high leaf to area ratio, specific leaf area, and chlorophyll concentration; low light compensation point, low dark respiration and high quantum yield [2]. Shade-avoider plants alter their morphology and phenology to “escape” shade by favoring growth in height, increasing internode length, petiole elongation and leaf insertion angles, reducing branching, and by accelerating leaf senescence and flowering [3,4]. Photoreceptors, signaling pathways and molecular mechanisms that elicit the shade tolerance syndrome are not known so far [5]. By contrast, the steps from signal perception to molecular changes and genetic regulation that elicit the shade-avoidance syndrome (SAS) have been well characterized. The molecular mechanisms of SAS responses have been intensively investigated and are relatively well understood. Downstream photoreceptor signaling involves the participation of specific targets of the light signaling pathway, together with the combined action of a number of plant hormones, including gibberelins, auxins, brassinosteriods and ethylene [6,7].

Under a plant canopy, irradiance is attenuated in a wavelength-selective manner. Plant leaves largely absorb ultraviolet radiation (UV, 280 a 400 nm) and photosynthetically active radiation (PAR, 400–700 nm), mainly in the red (R: 600–700 nm) and blue (B: 400–500 nm) regions of the spectrum and reflect and transmit far red (FR: 700–800 nm) radiation. Attenuation of light quantity (PAR) and changes in light quality are perceived by an array of plant photoreceptors: the UV-B-absorbing UVR8 photoreceptor, the UV-A /B- (315–500 nm) absorbing phototropins and cryptochromes, and the R- and FR-absorbing phytochromes. The red to far-red (R:FR, 660±10: 730±10 nm) ratio can be used by plants to detect, via phytochromes, direct sunlight exposure (R:FR = 1.1) or plant-shading (R:FR < 1.1). Phytochromes can also detect reductions in lateral R:FR ratios due to the proximity of neighboring plants, even before being over-shaded (without PAR attenuation). Phytochromes are chromo-proteins that interconvert between two molecular forms, the R-absorbing and biologically inactive form, Pr and the FR-absorbing and biologically active form, Pfr. Different proportions of Pr and Pfr stabilize at different R:FR values establishing a phytochrome photoequilibrium: Pfr/(Pfr+Pr). At low R:FR, the low ratio between Pfr/(Pfr+Pr) has been shown to trigger SAS responses [8]. Five phytochromes (phyA—E) have been described in *Arabidopsis thaliana*, of which phytochrome B (phyB) is the main sensor of R:FR in de-etiolated plants [9]. Plant species that display SAS responses commonly grow in relatively open habitats and respond to lateral low R:FR before over-shading [10–12]. In this scenario, SAS increases the possibilities of overtopping neighboring competitors to succeed in light foraging.

The morphological SAS response to low R:FR elicited by phytochrome B is also accompanied by growth, developmental and physiological responses that affect water relations and carbon economy. In *A*. *thaliana* and many herbaceous crops such as potato, tomato, bean, cucumber, cotton and rice, SAS responses are associated with reduced transpiration, photosynthesis, stomata density, stem hydraulic conductivity and soil exploration by roots. These traits confer tolerance to and/or evasion of water scarcity in these crop species, with the exception of *A*. *thaliana* phyB mutants, in which a lower ABA sensitivity decreases drought tolerance despite the abovementioned responses [13,14]. Thus, phytochrome B participates in the acclimation of the hydraulic architecture of shade avoider plants, adjusting water transport and carbon assimilation in concordance with the light environment, in anticipation of light *and* water competition with neighboring plants. In crop production, the responses of plants to the light environment affect crop yield and quality. High plant densities or self-shading inside a profuse canopy deviate, carbohydrates to stem growth, causing a detriment in crop yield of shade avoider plants [15–19]. Consequently, since vegetation shading alters yield and quality of harvestable organs, as well as the hydraulic architecture of crops, decisions on plant densities and canopy modeling by pruning or trellis systems (particularly important in fruit tree production) should also take into account that changes in the light environment could affect crop water use and drought tolerance.

*Vitis vinifera* (grapevine) is the most widely cultivated woody perennial species and economically important fruit crop in the world [20]. In arid and semiarid climates grapevine is cultivated under irrigation and is subjected to moderate water deficit during certain periods of fruit development to increase concentration of sugars, phenolics and volatile organic compounds, causing the detriment of vegetative growth [21,22]. The balance between positive (i.e. grape quality for winemaking) and negative (e.g. reduced photosyntesis, xylem cavitation) effects of water stress on grapevine production depends on how the irrigation schedule accounts for cultivar tolerance to water deficit. Vineyards are managed from the moment of establishment throughout their annual cycle with practices that affect both light quantity and quality at different levels within the canopy and around the fruits: plant density, row orientation, trellis system, pruning, shoot thinning and positioning, as well as leaf removal [23,24].

Past research work on grapevine response to light mainly focused on different PAR levels. For diverse varieties of *Vitis vinifera* L. (i.e., Cabernet Sauvignon, Cabernet Franc, Trebbiano Toscano, Muscat Gordo Blanco, Riesling, Syrah, Almeria, Sultanina, Sangiovesse, Chasselas) grown under decreasing PAR intensities, shoot and internode length, as well as individual leaf area, remained constant. However, stem diameter and leaf thickness were reduced, which in turn resulted in an increased in leaf area ratio. Lowering PAR increases grapevine quantum yield, and diminishes dark respiration and light compensation point [25–30]. Altogether these morphological and physiological grapevine traits suggest a shade tolerant acclimation response to low light intensity. Low PAR levels also altered xylem architecture and reduced hydraulic conductance of Riesling plants [28]. By contrast, we are only aware of one study that investigated *Vitis vinifera* response to variations in light quality within the visible and FR spectrum. Gonzalez et al. [31] demonstrated that R and B light incident on individual clusters of a commercial vineyard increased phenolic compounds in the skin of Malbec berries without affecting soluble solids, acidity or berry size. Taken together, these results indicate that *Vitis vinifera* acclimate to neutral shade (PAR attenuation without modification of the spectral composition) by maximizing light interception and use, and altering xylem architecture with an impact on water transport. In addition, results further suggest that light quality perception by fruit-localized photoreceptors enhances grape attributes for winemaking. The fact that light quality can modulate grape traits of central importance for high quality wines reveals that the responses of *Vitis vinifera* to the light environment are not only related to light quantity or temperature, but also to light spectral composition.

In the field, variations in PAR quantity due to cultural practices and common crop management decisions, are accompanied by variations in R:FR [32–34]. While the responses of vegetative organs of *Vitis vinifera* to neutral shade are well documented, their response to variations in R:FR have, by contrast, not been explored so far. In this study we aimed to investigate the morphological, biochemical and hydraulic responses of *Vitis vinifera* L. cv. Syrah and Torrontés Riojano cultivars to reductions in lateral R:FR to describe responses mediated by phytochromes. Given the evidences that grapevine vegetative organs demonstrate shade tolerant behavior to decreasing PAR, and that low PAR alters xylem architecture, reducing its hydraulic conductivity, we predict that low R:FR will induce changes in grapevine hydraulic architecture that confer tolerance to water restriction, together with a slight or null morphological SAS response.

## Material and Methods

### Experimental design and growth conditions

Dormant own-rooted cuttings of *Vitis vinifera* cv. Syrah and Torrontés Riojano were grown in 8 L pots filled with sand, in a glasshouse at Instituto de Biología Agrícola de Mendoza in Luján de Cuyo, Mendoza, Argentina (33°0′S, 68°52′W, 940 m asl). Plants were watered daily to field capacity with a drip irrigation system and fertilized weekly with 0.4 g L^-1^ of 18-18-18 NPK (9,9% NO_3_^-^ and 8,1% NH_4_^+^, 18% P_2_O_5_ and 18% K_2_O) and micronutrients (Red Hakaphos, Compo, Spain). One shoot was left to grow while the others were excised. Plants were supported by 2-m long, thin-wooden poles.

**Fig 1:**
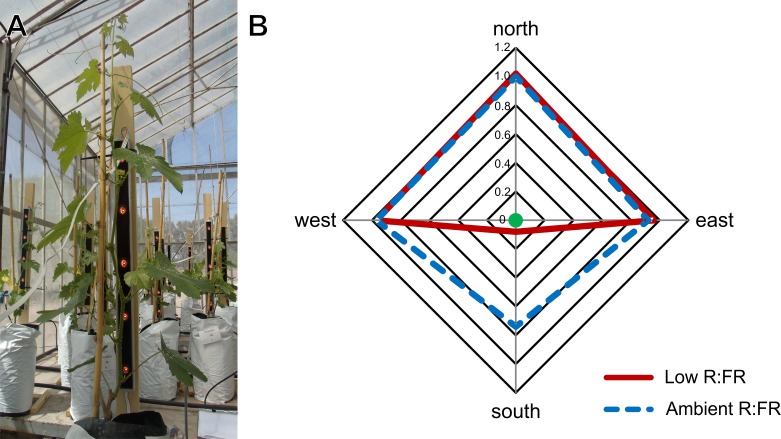
(A) Grapevine plants with FR light sources at their southern side (low lateral R:FR treatment), (B) R:FR ratios received by plants (green dot) at each cardinal point (north, east, south and west) in both light treatments: low (red) and ambient lateral R:FR (blue) at solar noon; n = 9. Lines are drawn for graphical clarity. Measurements were taken by placing a R:FR sensor head sideways facing towards the 4 cardinal points at module’s center height (See M&M for further details).

The experiment was a 2 × 2 factorial. We tested the behavior of two commercial grapevine varieties (V; Syrah and Torrontés Riojano) in two light treatments (T; low lateral vs. ambient R:FR). The experiment was designed as a completely randomized 2 × 2 factorial with 9 blocks along a northern-southern PAR gradient inside the glasshouse. Individual plants were the experimental units. The low lateral R:FR treatment was applied by lateral supplementation with FR (peak λ = 730 nm) light using Green Power LED research modules (Philips, Amsterdam, Netherlands) (Fig 1A). LEDs modules were mounted on a vertical wooden board placed 0.07 m away from plants at their southern side. Plants were irradiated during the natural photoperiod plus 1 h at the end of the day with 70 μmol m^-2^ s^-1^ of FR light. Duration of light treatment was controlled daily with digital timers which were programmed weekly according to photoperiod variation. For the ambient lateral R:FR treatment, wooden boards with black strips that simulated the presence of LED modules were placed in the southern side of the plants. To guarantee light treatments were correctly imposed, we characterized the light environment around each plant by measuring zenithal and lateral PAR and R:FR with Skye SKR110/116 hemispherical sensors attached to the SpectroSense +2 (Skye Instruments Ltd, Powys, UK). PAR and R:FR were measured by placing the sensors heads pointing towards the zenith (perpendicular to the ground surface) and sideways facing towards the 4 cardinal points at the center of the module’s height (0.8 m). Light measurements were taken at solar noon under clear sky conditions. Low lateral R:FR treatment was 0.07±0.003 (Fig 1B, S1 Fig).

Two consecutive experiments were set up in different periods: *i)* September 27 to December 3, 2013 (two-month-old plants); *ii)* December 4 2013 to April 4, 2014 (four-month-old plants). The second experiment was longer than the first one because the stem anatomical analysis required lignified tissues.

During the experimental period, mean midday PAR and UV-B radiation inside the glasshouse were 700±50 μmol m^-2^ s^-1^and 0.43±0.2 μW cm^-2^, respectively, since the glasshouse was covered by an anti-hail net (shade factor = 17%) and a shadecloth (shade factor = 30%). A PMA2200 radiometer with a PMA2102 UV-B detector (Solar Light Company Inc., Glenside, PA, USA) was used to measure UV-B radiation (280–315 nm). Mean daily temperature and relative humidity (RH) inside the glasshouse were 23±5°C and 37±20% respectively, measured every 30 min with continuous sensors (HOBO, Pro Series data loggers, Onset Computer Corporation, Bourne, MA, USA).

### Growth and morphology

Stem length, number of nodes and number of axillary lateral shoots were recorded weekly. At the end of the experiments, petiole length, leaf angle of fully expanded basal leaves (except for first and second leaves), internode length and shoot basal diameter were measured. Petiole length, leaf angle and internode length are reported as an average of three measurements per plant. Plant organs were separated into leaves, axillary shoots and main stem for leaf area (LA) and biomass determinations. LA was measured with a portable LI-3100C Area Meter (LI-COR Biosciences, Lincoln, Nebraska, USA). To determine biomass, plant material was dried at 65°C for 48 h, and then weighed. Specific leaf weight (SLW, mg cm^-2^) was calculated by weighing ten leaf discs of 1 cm^2^ per leaf. SLW is reported as an average of the SLW of three leaves per plant.

### Biochemical measurements

**Plant hormones:** samples were taken at dusk (sunset) in order to avoid photodegradation of IAA and ABA. Three expanded leaves per plant (up to LED module’s height) were pooled and collected in liquid nitrogen. Leaf samples were then lyophilized and ground to powder with mortar and pestle, and aqueous extractions were taken to determine GA_1_, GA_3_, IAA and ABA concentrations. Hormone identification and quantification was done by liquid chromatography and mass spectrometry, as described in Masciarelli et al. [35] at the Plant Physiology Laboratory, National University of Rio Cuarto, Córdoba, Argentina.

**Photosyntetic and photoprotective pigments:** chlorophylls and carotenoid contents were determined in leaves (sampled up to LED module’s height), after extraction of 1 cm^2^ of leaf sample in 5 mL of dimethyl sulfoxide at 70°C in darkness for 45 min. Absorbance at 665, 649 and 480 nm (UV-vis Spectrum SP-2000; Shangai, China) was measured according to Chapelle *et al*. [36]. The equations of Wellburn [37] were used to calculate chlorophyll *a*, *b* and carotenoids. The UV-absorbing phenolic compounds (UVAC) and anthocyanin content in leaves (sampled up to LED module’s height) were determined after extraction of 5 cm^2^ of leaf sample in 10 mL HCl-methanol (1% w/v) at -18°C for 48 h and absorbance was measured at 280 and 546 nm (UV-vis spectrophotometer). Pigments were expressed on a dry weight and leaf area basis.

### Water-related traits

**Plant hydraulics:** Transpiration rate per leaf area unit leaf area (E) was estimated by weighing pots enclosed in bags (to prevent substrate evaporation) at 1 h-intervals around solar noon, and expressed in a LA basis as Kg H_2_O m^-2^ s^-1^. Stomatal conductance (g_s_) was measured with a steady-state diffusion porometer (SC-1, Decagon Devices, Pullman, WA, USA) on the abaxial leaf surface of fully expanded leaves (up to LED module’s height) at solar noon, and expressed as mmol of air m^-2^ s^-1^. Ambient conditions during transpiration rate and stomatal conductance measurements were PAR = 725 μmol m^-2^ s^-1^, air temperature = 28°C and RH = 35%. Plant hydraulic conductance (K_plant_) was calculated as the ratio between E and the water potential gradient (ΔΨ) between plant and soil, expressed in a LA basis as Kg s^-1^ MPa^-1^ m^-2^. Pre-dawn (Ψ_PD_) and midday (Ψ_MD_) leaf water potential was measured according to Scholander et al. [38] with a pressure chamber (Modelo 4, BioControl, Buenos Aires, Argentina). Ψ_PD_ and Ψ_MD_ were determined at 3:00 a.m. and at solar noon, respectively. Ψ_PD_ was used to estimate soil water potential. The night before measuring Ψ_PD_, plants were covered with black polyethylene bags to prevent nighttime transpiration.

Maximum stem hydraulic conductivity (k_s max_) of basal woody stem segments (0.7–0.9 m length) was determined as described in Fernández and Gyenge [39]. Leaves and lateral shoots were removed and exposed surfaces were sealed with instantaneous glue (LA GOTITA®; ALKAPOL SA, Buenos Aires, Argentina). Stems were then cut, submerged in water and connected to a pressurized water source (1.7 Bar) for 5 min to eliminate embolisms. k_s max_ was expressed as Kg s^-1^ m^-1^ MPa^-1^ and calculated with the following equation:

$$k_{s\;\max}=\frac{Q\;l}{A\;P}$$ (1)

where *Q* is water flow rate (Kg s^-1^) calculated from the mass of water that passes through the stem segment in 1 min, *l* (m) is stem segment length, *A* (m^2^) is the average transversal area of both ends of the stem segment excluding the bark, and *P* (MPa) is the pressure applied to the system. For the leaf elastic response and osmoregulation, pressure-volume (P-V) curves were obtained from fully-expanded leaves (up to LED module’s height) by measuring relative water content (RWC) at different values of leaf Ψ [40]. Leaves were kept for 12 h in plastic bags with their cut ends in distilled water, in order to reach full turgor. Subsequent paired measurements of Ψ (measured as described above) and weight were done as leaves transpired freely in the laboratory. After measurements, plant material was oven-dried at 65°C for 48 h to calculate RWC. We fitted Ψ vs. RWC curves with the Pressure-Volume Analysis Programme developed by Schulte and Hinckley [41], and calculated osmotic potential at full turgor (Ψs), osmotic potential at turgor loss point (Ψs_tlp_), relative water content at turgor loss point (RWC_tlp_), symplastic fraction (Vs), and bulk modulus of elasticity (E_max_).

For stomata density and index, two epidermal imprints (abaxial leaf surface) per fully-expanded leaf (one leaf per plant sampled up to LED module’s height) were taken with transparent nail varnish. Five photographs per imprint were shot using a Micrometrics 318 CU camera (Beijing, China) attached to a Nikon Eclipse E200 optical microscope (Tokyo, Japan) at 400 magnification. Stomatal density (SD, number of stomata per unit leaf area) and stomata index (SI, ratio between number of stomata and number of epidermal cell) were calculated.

At the end of the experiment, wood anatomy was assessed on woody stem portions located between the fourth and fifth internodes. After cutting, samples were kept in FAA buffer (ethanol 96% v/v: distilled water: formaldehyde: acetic acid, 50:35:10:5) before transverse thin microsections of 16–18 μm were prepared with a sliding microtome (Euromex Microscopen B.V., As Arnhem, Holland). Microsections were stained with safranine (1% w/v) and astrablue (0.5% w/v), rinsed with distilled water and successive solutions of increasing ethanol concentration (50, 75 and 100% v/v) and xylol, permanently mounted on microscope slides with Canada balsam before they were oven dried at 65°C for 24 h. Microsections were observed at 25× magnification under a light microscope (DM2000, Leica Microsystems, Heerbrugg, Switzerland) equipped with a digital camera (DFC 320, Leica Microsystems). We measured xylem cross-sectional area (XA), number (NV) and density (VD) of vessels, vessel lumen area (TLVA), average lumen vessel area (ALVA) and both lumen diameters (ALVD_max_: maximum average lumen vessel diameter and ALVD_min_: minimum average lumen vessel diameter) with the software WinCELL Pro V 2004a (Regent Instruments Inc., Canada) following the procedures described in Arbellay et al. [42,43]

### Statistical analysis

All statistical tests were done with Infostat 2011 software [44]. Data were analyzed by fitting linear mixed-effects models, considering variety (V), treatments (T) and their interactions (VxT) as fixed factors, and blocks (B) as a random factor; α = 0.05. Selected models were tested for homoscedasticity and normality of residuals by visual assessment of plots. The correct variance structure used in the fitted models was determined by comparison of Akaike’s and Bayesian’s Information Criterion. The models for leaf DW, stem DW, total shoot biomass and plant hormone concentrations (AG_3_, AG_1_ and auxin) were fitted by the addition of the varIdent variance structure to the random part of the model. Repeated measures in time of stem length and number of nodes were analyzed considering growth days (D) and their interactions (TxD, DxV and VxTxD) as additional fixed factors. The functions varldent and corSymm were used to specify an unstructured covariance matrix for both variables. Post-hoc comparison of means was done with DGC multiple-comparisons test [45].

Power analysis calculations were done with G*Power 3.1 software [46]. For sample size estimation, a statistical power analysis was done based on data from a pilot study carried out on *Solanum lycopersicum* cv. Money Maker. Stem length of one-month old plants (eight fully expanded leaves,) grown under similar growth conditions to those described in this study, was measured (N = 28). Ambient (mean = 26.79 and SD = 2.59) to low lateral R:FR (mean = 59 and SD = 1.49) treatments were compared. The effect size (ES) in this study (F-test, post-hoc, ANOVA: fixed effects, one-way) was f = 7.63, considered to be extremely large using Cohen's (1988) criteria. Considering an ES = 7.63, with α = 0.05, power = 0.80 and 4 number of groups, the projected total sample size needed for our experimental design was N = 5 (F-test, a priori, ANOVA: main effects and interactions). Thus, our proposed total sample size of 36 was more than adequate for the main objective of this study as the power (1 –beta) was equal to 1.

In addition, to determine the ES that we could detect with our experimental design, a sensitivity analysis was performed (F-test, sensitivity, ANOVA: main effects and interactions). Considering α = 0.05, power = 0.80, 4 groups and N = 36, we were able to detect effects higher than 0.48 (considered large according to Cohen’s criteria).

## Results

**Fig 2:**
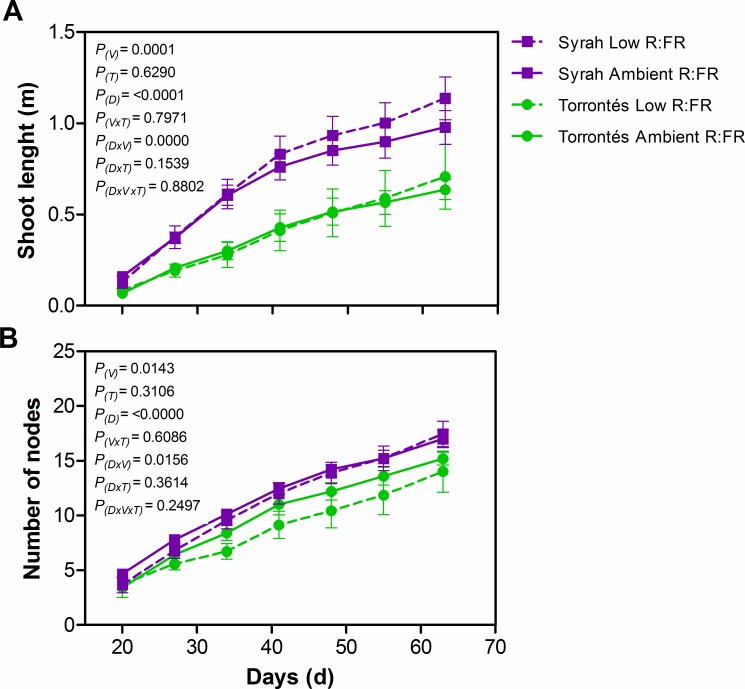
Stem growth of two-month-old grapevine plants grown under different lateral R:FR ratios. (A) Shoot length and (B) number of nodes per shoot in time. Values are means ± SE, n = 9. *P-*values of variety (*P*_(V)_), treatment (*P*_(T)_), days of growth (*P*_(D)_), variety x treatment interaction (*P*_(V×T)_), days of growth x variety interaction (*P*_(D×V)_), days of growth x treatment interaction (*P*_(D×T)_) and days of growth x variety x treatment interaction *P*_(D×V×T)_ are reported; df = 18.

**Fig 3:**
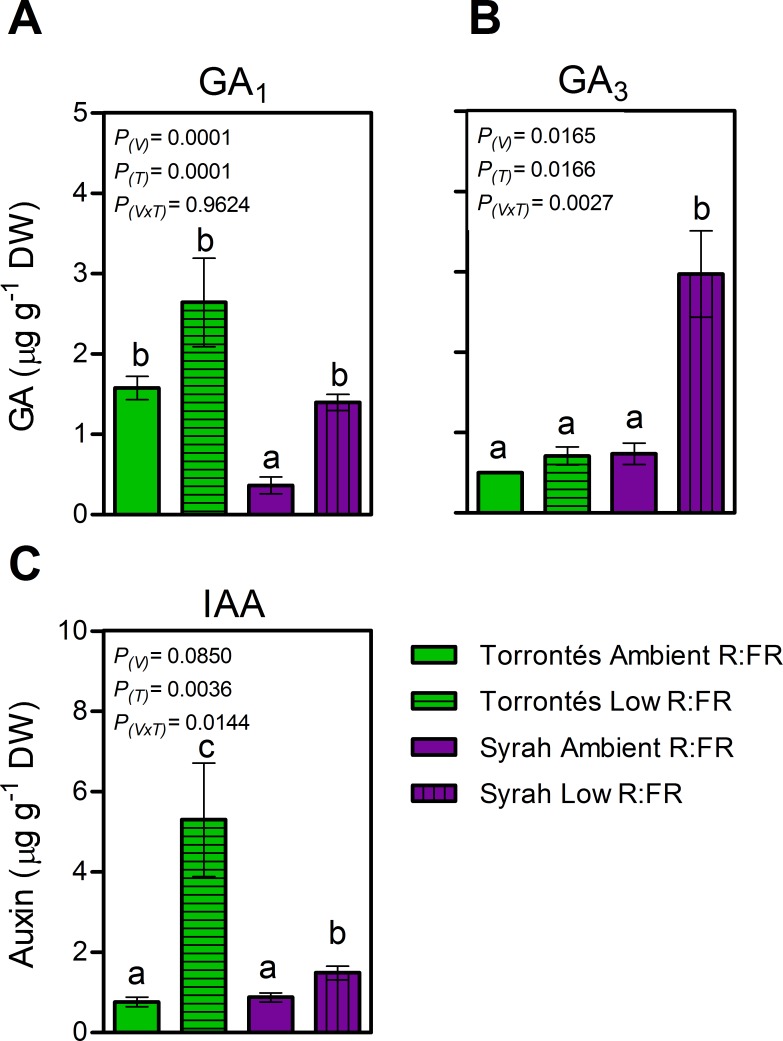
Growth-related phytohormones concentrations measured in leaf-tissue of two-month-old grapevine plants grown under different lateral R:FR ratios. (A) Gibberellic acid 1, (B) Gibberellic acid 3, (C) Indol acetic acid. Values are means ± SE, n = 5. *P-*values of variety (*P*_(V)_), treatment (*P*_(T)_) and variety x treatment interaction (*P*_(V×T)_) are reported, df = 10.

**Fig 4:**
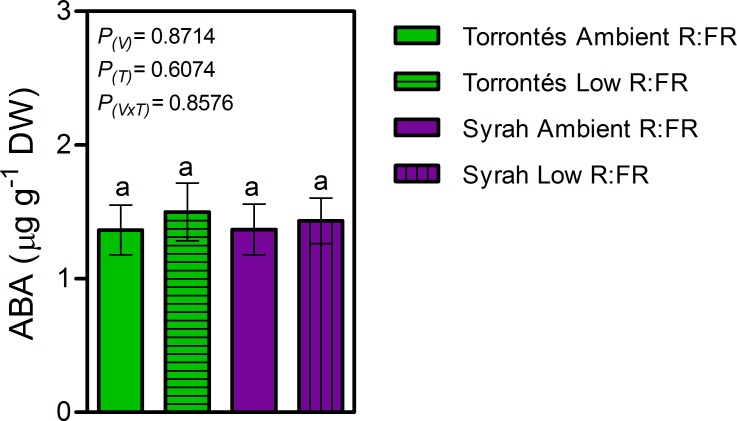
ABA concentration in leaf-tissue of two-month-old grapevine plants grown under different lateral R:FR ratios. Values are means ± SE, n = 5. *P-*values of variety (*P*_(V)_), treatment (*P*_(T)_) and variety x treatment interaction (*P*_(V×T)_) are reported, df = 10.

Syrah plants were taller than Torrontés Riojano’s (Fig 2). In both varieties, the main stems did not alter significantly in length, number of nodes, diameter, internode length, or number of lateral shoots in response to low lateal R:FR (Fig 2 and Table 1). Leaves of both varieties were non-responsive to low lateral R:FR, and maintained similar petiole length and angle, total LA per plant, individual stem LA, LA per axillary shoot, and SLW in both light treatments (Table 1). Total plant biomass and biomass allocation among leaves, main stem and lateral shoots biomass were not affected by low lateral R:FR (Table 1). In that sense, Syrah and Torrontés Riojano plants did not display any of the typical SAS morphological markers in response to a lateral reduction in R:FR. Despite this lack of significant morphological and growth responses, low lateral R:FR increased leaf concentration in hormones participating in cellular division and elongation, namely GA_3_ and GA_1_ in Syrah, and IAA in Syrah and Torrontés Riojano (Fig 3). The latter demonstrated a variety-specific biosynthesis of growth-hormones in response to the light treatments. Experiments also revealed that leaf ABA concentration was similar in both light treatments (Fig 4).

**Table 1:** Morphology and growth traits.

Variables	Torrontés Ambient R:FR	Torrontés Low R:FR	Syrah Ambient R:FR	Syrah Low R:FR	P_(V)_	P_(T)_	P_(V×T)_	df
**Shoot length^1^ (cm)**	63.6±5.5	70.7±17.8	97. 8±9.3	113.7±11.7	0.0073	0.3787	0.7346	18
**Number of nodes^1^**	15.2±0.6	14.0±1.8	17.0±0.8	17.4±1.1	0.0043	0.8719	0.5243	18
**Internode length^1^ (mm)**	42.4±5.3	43.1±0.9	57.8±5.0	68.1±8.2	0.0176	0.4004	0.5275	17
**Shoot diameter^1^ (mm)**	3.9±0.2	4.4±0.3	4.3±0.3	4.6±0.3	0.5078	0.2363	0.8182	17
**Axillary shoots per plant**	4.4±1.0	4.3±1.2	7.6±0.6	7.4±0.7	0.0048	0.9090	0.9987	18
**Petiole length (mm)**	7.1±0.4	7.4±0.5	6.8±0.3	7.3±0.3	0.6564	0.2589	0.8770	18
**Leaf angle (degrees)**	55.5±4.9	56.9±2.4	58.7±2.3	57.1±2.0	0.4599	0.8969	0.6267	18
**SLW (mg DW cm^-2^)**	4.2±0.1	4.4±0.2	4.5±0.5	4.5±0.8	0.4846	0.8170	0.5604	18
**LA per plant (m^2^)**	0.26±0.01	0.28±0.03	0.30±0.01	0.28±0.01	0.2945	0.6328	0.2878	9
**Individual LA (cm^2^)**	163±6	163±4	120±8	110±7	0.0001	0.4452	0.5462	9
**Axillary shoot LA (cm^2^)**	430±70	548±169	680±93	640±81	0.0931	0.8490	0.3987	9
**Stem DW (g)**	8±0.8	11±2.9	16.3±1	14±0.9	0.0004	0.4492	0.0837	9
**Leaf DW (g)**	13.4±0.8	16.9±4.3	15.8±0.9	13.8±0.8	0.9666	0.2532	0.1411	9
**Lateral shoot DW (g)**	2.7±0.6	3.6±0.6	4.1±0.5	3.7±0.5	0.1476	0.8216	0.2676	9
**Total shoot biomass (g)**	24.2±2	32.2±6.7	36.1±1.9	31.6±1.9	0.0454	0.4070	0.0594	9

Vegetative growth measurements of two-month-old grapevines grown under different lateral R:FR ratios.

Values are means ± SE, n = 9. *P*-values of variety *(P*_(V)_), treatment (*P*_(T)_)_,_ and variety × treatment interaction (*P*_(V×T)_) are reported. SLW, specific leaf weight; df, denominator degrees of freedom.

^1^ Variables measured in main shoot.

Chlorophyll *a* and *b*, total chlorophylls, carotenoids, chlorophyll:carotenoid ratio, UV- absorbing phenolic compounds and anthocyanins expressed per unit of LA and per leaf DW remained unaffected by low lateral R:FR in both varieties (Table 2). Therefore, reduced R:FR ratios did not affect traits related to photosynthesis and photoprotection.

**Table 2:** Photosynthesis related traits.

Variables	Torrontés Ambient R:FR	Torrontés Low R:FR	Syrah Ambient R:FR	Syrah Low R:FR	P _(V)_	P _(T)_	P _(V×T)_	df
**Chl a (μg mg**^**-1**^**)**	1.24±0.13	1.25±0.07	1.09±0.06	1.26±0.15	0.5049	0.3594	0.4947	18
**Chl b (μg mg**^**-1**^**)**	0.48±0.05	0.50±0.03	0.42±0.03	0.48±0.06	0.3860	0.2900	0.5853	18
**Total Chl (μg mg**^**-1**^**)**	1.7±0.2	1.7±0.1	1.5±0.1	1.7±0.2	0.4690	0.3382	0.5186	18
**Car (μg mg**^**-1**^**)**	0.25±0.02	0.25±0.01	0.23±0.01	0.25±0.02	0.5016	0.5056	0.6938	18
**Chl a (ng mm**^**-2**^**)**	49.3±5.2	50.2±2.1	45.8±1.7	52.7±3.7	0.8458	0.1720	0.3632	18
**Chl b (ng mm**^**-2**^**)**	19.0±2.1	19.9±0.9	17.5±0.7	20.3±1.5	0.6260	0.1234	0.4887	18
**Total Chl (ng mm**^**-2**^**)**	68.4±7.3	70.1±2.9	63.3±2.3	72.9±5.1	0.7794	0.1553	0.3955	18
**Car (ng mm**^**-2**^**)**	9.9±0.8	10.1±0.2	9.7±0.3	10.5±0.5	0.9186	0.2396	0.5842	18
**TChl: Car**	6.7±0.2	6.9±0.2	6.5±0.1	6.9±0.2	0.3711	0.1902	0.3989	18
**UVAC (OD**_**305nm**_ **mg**^**-1**^**)**	0.33±0.03	0.28±0.05	0.29±0.03	0.35±0.03	0.4364	0.5229	0.0873	18
**UVAC (OD**_**305nm**_ **cm**^**-2**^**)**	1.1±0.1	0.9±0.1	1.0±0.1	1.2±0.1	0.5337	0.5163	0.2688	18
**Anthocyanins (OD**_**546nm**_ **mg**^**-1**^**)**	0.02±2 E-3	0.02±3 E-3	0.02±2 E-3	0.02±2 E-3	0.2133	0.5719	0.0556	18
**Anthocyanins (OD**_**546nm**_ **cm**^**-2**^**)**	0.07±4 E-3	0.07±0.01	0.07±4 E-3	0.08±0.01	0.2318	0.5113	0.1873	18
**SD (stomata mm**^**-2**^**)**	228.5±12	193.1±12	152.6±8	156.0±10	0.0001	0.2720	0.0862	18
**SI**	0.09±0.01	0.08±2 E-3	0.06±3E-3	0.06±2E-3	0.0001	0.9669	0.3561	18

Photosynthetic and photoprotective pigments and morphological traits associated to photosynthetic process in leaves of two-month-old grapevines grown under different lateral R:FR ratios. Chl a, chlorophyll a; Chl b, chlorophyll b; TChl, total chlorophylls; Car, carotenoids; UVAC, UV radiation absorbing compounds (OD_305 nm_); anthocyanins (OD_546nm_); SD, stomata density; SI, stomata index. Values are means ± SE, n = 9. *P*-values of variety (*P*_(V)_), treatment (*P*_(T)_)_,_ and variety × treatment interaction (*P*_(V×T)_) are reported; df, denominator degrees of freedom.

Low lateral R:FR did not alter transpiration, stem hydraulic conductivity or whole plant water transport capacity (K_plant_), as shown in Table 3. Diurnal patterns of stomata conductance remained unaffected by reduced lateral R:FR as well (only data at solar noon is presented in Table 3), as did stomata density or the stomata index (Table 2). This lack of effect on water loss and water transport capacity was accompanied by similar xylem cross-sectional area, number of vessels, number of conduits per xylem area and minimum average lumen vessel diameter in both light treatments. Maximum average lumen vessel diameter was higher under Ambient R:FR than under low lateral R:FR in both varieties (the difference was 3 and 5 μm in Torrontés and Syrah respectively; *P*_T_ = 0.0297). However this change in dimension did not affect average lumen vessel area (Table 4). As a consequence, maximum stem hydraulic conductivity was not significantly different among treatments or varieties. This also means that water use, transport and hydraulic architecture of Syrah and Torrontés Riojano were not altered by low lateral R:FR. None of the classical parameters of the P-V curve–i.e. Ψs, Ψs_tlp_, RWC_tlp_, Vs and Emax—were affected by variations in R:FR (Table 5), indicating that light treatments did not alter leaf water relations such as osmoregulation or cell wall elasticity.

**Table 3:** Water relations.

**Variables**	Torrontés Ambient R:FR	Torrontés Low R:FR	Syrah Ambient R:FR	Syrah Low R:FR	P_(V)_	P_(T)_	P _(V×T)_	df
**Ψ_PD_ (MPa)**	-0.43±0.02	-0.43±0.02	-0.37±0.03	-0.34±0.04	0.0540	0.5172	0.6014	18
**Ψ_MD_ (MPa)**	-0.82±0.03	-0.80±0.02	-0.80±0.02	-0.77±0.03	0.3918	0.2635	0.9203	18
**E (kg H_2_O m^-2^ s^-1^)**	2.8E-5±4E-6	3.3E-5±2E-6	3.5E-5±6E-6	1.8E-5±5E-6	0.7083	0.5837	0.3452	18
**K_plant_ (kg s^-1^ MPa^-1^ m^-2^)**	7.3E-5±1E-5	1E-4±1E-5	7.6E-5±1E-5	3.9E-5±9E-6	0.2309	0.9510	0.0605	17
**g_s_ (mmol m^-2^ s^-1^)**	481±33	448±49	430±47	431±37	0.4063	0.7872	0.7137	18
**k_s max_ (kg s^-1^ m^-1^ MPa^-1^)**	5.9±0.5	7.1±0.5	9.6±0.7	8.7±0.7	0.0035	0.8551	0.1502	18

Physiological traits related to water use in two-month-old grapevines plants grown under different lateral R:FR ratios. Ψ_PD_, pre-dawn water potential; Ψ_MD_, midday water potential; E, transpiration rate; K_plant_, plant conductance; g_s_, stomatal conductance; k_s max_, maximum stem hydraulic conductivity. Values are means ± SE, n = 9. *P*-values of variety (*P*_(V)_), treatment (*P*_(T)_)_,_ and variety × treatment interaction (*P*_(V×T)_) are reported; df, denominator degrees of freedom.

**Table 4:** Woody anatomy.

Variables	Torrontés Ambient R:FR	Torrontés Low R:FR	Syrah Ambient R:FR	Syrah Low R:FR	*P*_(V)_	*P*_(T)_	*P*_(V×T)_	df
**XA (mm^2^)**	32.3±1.2	30.3±1.8	28.9±2.6	29.5±1.8	0.3990	0.8950	0.5962	8
**NV**	1379±27	1489±69	1253±78	1296±58	**0.0238**	0.2538	0.3697	8
**VD (vessels mm^-2^)**	89±6	87±6	75±1	85±5	0.2135	0.2540	0.2605	8
**TLVA (mm^2^)**	15.4±1.2	13.1±1.2	29.5±16.7	14.4±0.9	0.5261	0.3282	0.5633	8
**ALVA (μm^2^)**	2172±222	1966±216	2601±49	2220±128	0.0613	0.0563	0.5768	8
**ALVD_max_ (μm)**	42.6±1.8	39.7±2.0	46.18±0.6	41.9±1.5	0.1094	**0.0297**	0.6568	8
**ALVD_min_ (μm)**	40.6±2.3	39.2±2.6	46.6±0.6	41.4±1.7	0.0652	0.0557	0.3221	8

Stem wood anatomy analysis in four-month-old grapevine grown under different lateral R:FR ratios.

XA: xylem cross-sectional area, NV: total number of vessels, VD: vessel density, TLVA: total lumen vessel area, ALVA: average lumen vessel area, ALVD_max_: maximum average lumen vessel diameter, ALVD_min_: minimum average lumen vessel diameter. Values are means ± SE, Syrah: n = 6 and Torrontés: n = 3. *P*-values of variety (*P*_(V)_), treatment (*P*_(T)_)_,_ and variety × treatment interaction (*P*_(V×T)_) are reported; df, denominator degrees of freedom.

**Table 5:** P-V Curves.

*Variables*	Torrontés Ambient R:FR	Torrontés Low R:FR	Syrah Ambient R:FR	Syrah Low R:FR	*P*_(V)_	*P*_(T)_	*P*_(V×T)_	df
**Ψs (MPa)**	-1.4±0.09	-1.35±0.07	-1.42±0.04	-1.38±0.12	0.6802	0.6861	0.4512	15
**Ψs**_**tlp**_ **(MPa)**	-1.76±0.06	-1.73±0.10	-1.81±0.06	-1.97±0.13	0.6351	0.2209	0.9875	15
**RWC**_**tlp**_ **(%)**	0.87±0.01	0.88±0.01	0.83±0.02	0.87±0.01	0.0751	0.0547	0.3248	15
**Vs**	0.63±0.07	0.58±0.05	0.67±0.07	0.69±0.12	0.8696	0.2760	0.4515	15
**E_max_**	-16.9±1.8	-14.8±0.7	-17.1±2.9	-13.7±1.3	0.8329	0.1398	0.7801	15

Pressure-volume curves analysis in leaves of two-month-old grapevine plants grown under different lateral R:FR ratios.

Ψs, osmotic potential at full turgor; Ψs_tlp_, osmotic potential at turgor loss point; RWC_tlp_, relative water content at turgor loss point; Vs, symplastic fraction; E_max_, bulk elasticity modulus. Values are means ± SE, n = 5. *P*-values of variety (*P*_(V)_), treatment (*P*_(T)_)_,_ and variety × treatment interaction (*P*_(V×T)_) are reported; df, denominator degrees of freedom.

## Discussion

In this study, we demonstrated that the morphology and hydraulic architecture of *Vitis vinifera* cv. Syrah and Torrontés Riojano plants were unresponsive to strong reductions in lateral R:FR perceived by phytochromes. The sensitivity of our experimental setup enabled the detection of an effect size approximately 15 times lower than expected in standard experiments with low R:FR (see Materials and Methods for details). Consequently, whatever difference that was not detected by our statistical analysis, should be minimal with respect to reported data, and could be considered biologically irrelevant.

We found that Syrah and Torrontés Riojano plants did not alter their morphology in response to reduced lateral R:FR. The maintenance of stem height, internode length, leaf area, and biomass distribution between stem and leaf, indicates that these varieties did not display the classical SAS responses triggered by phytochromes in low lateral R:FR (Fig 2 and Table 1). If these results are taken together with previous r reports that documented that a wide range of grapevine varieties tend to “tolerate shade” at decreasing PAR (high leaf area ratio, specific leaf area, and chlorophyll concentration; low light compensation point, low dark respiration and high quantum yield) [25–30], we are submitting more evidence that suggests that *V*. *vinifera* might behave more like a shade tolerant than a shade avoider species. Shade avoidance and shade tolerance responses to low light are usually associated to the natural environment where plant species complete their life cycle: the former evolved in species that colonize open habitats and the latter in species that grow in the shade of higher vegetation strata [10,11]. Grapevine grows naturally in deciduous and semi deciduous Mediterranean forests [47], emerging in the understory and climbing trees up to the overstory [48]. The fact that this species is nowadays cultivated under high light intensity, demonstrate a high light-acclimation plasticity that might reflect this species’ ability to live under contrasting light environments. The strategies that plants employ to deal with shade may not be associated to a life form, since lianas SAS responses seem to be species-specific. Research conducted on woody vines from tropical, subtropical and temperate forests, have demonstrated that some species displayed SAS in response to low lateral R:FR while others did not.[49–51].

Although the varieties tested in our experiments did not elongate their stems, leaf concentration of growth-related hormones (GA and IAA) increased in response to low lateral R:FR (Fig 3). Similar variations in these hormone levels accompanied SAS response in shade-intolerant species such as Arabidopsis, tomato, maize, and rice [6,52,53]. The fact that hormones involved in SAS via phytochrome B action were increased in leaves with low lateral R:FR without concomitant internode and stem elongation, suggests that their sensitivity, transport or some action must be impeded somewhere downstream in the synthesis of GA and IAA. In this sense, *Stellaria longpipes* ecotypes that are sensitive (prairie habitats) and insensitive (alpine habitats) to low R:FR produce high levels of GA_1_ but different levels of GA_8_, the inactive product of the B-hidroxilation of GA_1_. Higher levels of GA_8_ in alpine ecotypes might explain their lack of stem elongation at low R:FR [53]. However, further research will be needed to elucidate the molecular mechanism involved behind the results found in this study.

Whole plant, leaf and xylem water transport capacity were not affected by reduced lateral R:FR, indicating that plant hydraulics was insensitive to light quality perceived by phytochromes in both varieties assayed. On the other hand, Schultz and Matthews [28] demonstrated that an 80% reduction in PAR altered xylem architecture and reduced its hydraulic conductance. Thus, *Vitis vinifera* hydraulic architecture seems more sensitive to low PAR levels than to reduced R:FR. More research must be conducted to elucidate how low-light hydraulic adjustment is regulated.

SAS responses are undesirable traits in crops, since plants divert a higher proportion of photoassimilates to the stem, in detriment of photosynthate partition to flowers or fruits, generally with negative consequences on yield [54]. If grapevine shoots do not evoke SAS in response to light quality perceived by phytochromes, canopy management practices underpinning changes in the R:FR will not deviate carbohydrates to stem growth in detriment of flower or fruit development and will not affect plant water relations. This, together with *Vitis vinifera* capacity to tolerate low PAR levels that permit a degree of shading without a proportional limitation on growth and yield [25], encourages revision of crop management decisions that modify vegetative growth. However, the revision must take into account an adequate sun exposure for cluster and fruitful buds to maintain high quality grape attributes and bud fruitfulness [55,56].

## Conclusions

In response to low lateral R:FR, grapevine plants increased concentration of growth-active gibberellins and auxins in leaf tissues, however they lack the classical morphological responses associated to SAS. This suggests that grapevines do not behave as a typical shade avoider species. Low lateral R:FR did not affect carbon allocation or the hydraulic architecture of plants. These findings encourage the revision of current canopy management practices that underpin changes in the R:FR ratio, since it does not have an effect on grapevine morphology, water relations and carbon economy.

## Supporting Information

S1 Fig Light environment of grapevine plants. (A) R:FR and (B) PAR received by plants from the zenith and from each cardinal point in both light treatments: low and ambient lateral R:FR; n = 9. PAR and R:FR were measured by placing the sensors pointing towards the zenith and sideways facing towards the 4 cardinal points at the center of module’s height (0.8 m). Data presented corresponds to measurements taken on October 18, 2013 at solar noon. (C) Light spectrum of the FR LED modules. The spectral scan of the FR light source (peak λ = 730 nm) was done with an USB4000 spectroradiometer using an optical probe with a CC-3-UV cosine corrector (Ocean Optics Inc., Dunedin, FL, USA).
(TIF)

S1 File Raw data supporting figures and tables. (XLSX)

## Abbreviations

B: blue
D: day/s
df: denominator degrees of freedom
DW: dry weight
E_max_: bulk elasticity modulus
FR: far red
k_s max_: maximum stem hydraulic conductivity
K_plant_: plant hydraulic conductance
LA: leaf area
no.: number
PAR: photosynthetic active radiation
phyB: phytochrome B
R: red
RWC_tlp_: relative water content at turgor loss point
SAS: shade avoidance syndrome
SD: stomata density
SI: stomatal index
SLW: specific leaf weight
UV: ultra violet
UVAC: UV-absorbing compounds
V: variety/ies
Vs: symplastic volume
Ψ: water potential
Ψs: osmotic potential
