# Supplementary figures and images for: Morphology and Hydraulic Architecture of Vitis vinifera L. cv. Syrah and Torrontés Riojano Plants Are Unaffected by Variations in Red to Far-Red Ratio
Source: PLoS One. 2016 Dec 2;11(12):e0167767. doi: 10.1371/journal.pone.0167767 (PMC5135135; doi:10.1371/journal.pone.0167767)

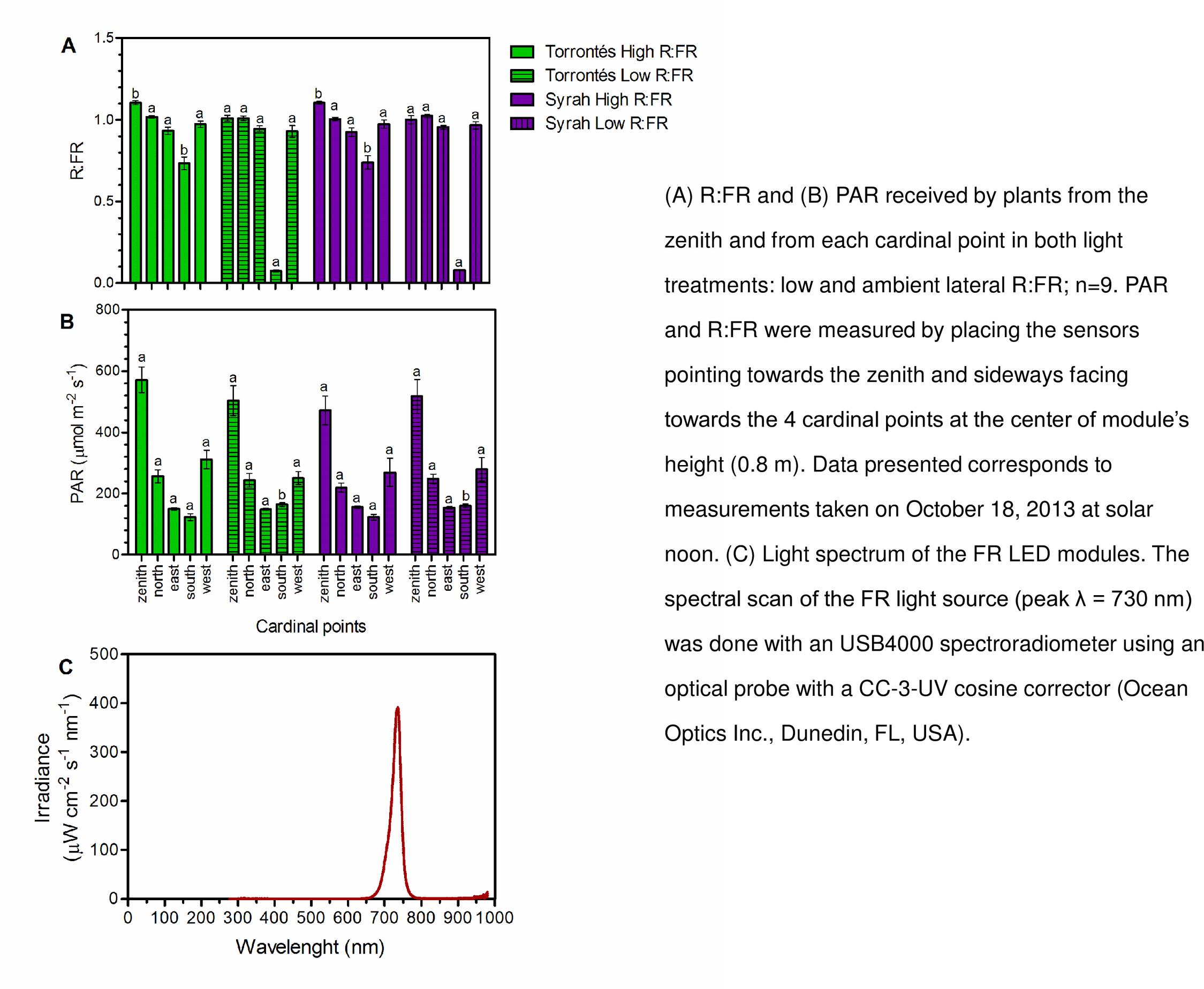

Supplement: S1 Fig — (A) R:FR and (B) PAR received by plants from the zenith and from each cardinal point in both light treatments: low and ambient lateral R:FR; n = 9. PAR and R:FR were measured by placing the sensors pointing towards the zenith and sideways facing towards the 4 cardinal points at the center of module’s height (0.8 m). Data presented corresponds to measurements taken on October 18, 2013 at solar noon. (C) Light spectrum of the FR LED modules. The spectral scan of the FR light source (peak λ = 730 nm) was done with an USB4000 spectroradiometer using an optical probe with a CC-3-UV cosine corrector (Ocean Optics Inc., Dunedin, FL, USA). (TIF) [file pone.0167767.s001.tif]
